# Cell Fate Decisions in the Wake of Histone H3 Deposition

**DOI:** 10.3389/fcell.2021.654915

**Published:** 2021-04-20

**Authors:** Reuben Franklin, Jernej Murn, Sihem Cheloufi

**Affiliations:** Department of Biochemistry, Stem Cell Center, University of California, Riverside, Riverside, CA, United States

**Keywords:** chromatin, histone H3, nucleosome diversity, histone chaperone, cell cycle, reprogramming, development, cellular plasticity

## Abstract

An expanding repertoire of histone variants and specialized histone chaperone partners showcases the versatility of nucleosome assembly during different cellular processes. Recent research has suggested an integral role of nucleosome assembly pathways in both maintaining cell identity and influencing cell fate decisions during development and normal homeostasis. Mutations and altered expression profiles of histones and corresponding histone chaperone partners are associated with developmental defects and cancer. Here, we discuss the spatiotemporal deposition mechanisms of the Histone H3 variants and their influence on mammalian cell fate during development. We focus on H3 given its profound effect on nucleosome stability and its recently characterized deposition pathways. We propose that differences in deposition of H3 variants are largely dependent on the phase of the cell cycle and cellular potency but are also affected by cellular stress and changes in cell fate. We also discuss the utility of modern technologies in dissecting the spatiotemporal control of H3 variant deposition, and how this could shed light on the mechanisms of cell identity maintenance and lineage commitment. The current knowledge and future studies will help us better understand how organisms employ nucleosome dynamics in health, disease, and aging. Ultimately, these pathways can be manipulated to induce cell fate change in a therapeutic setting depending on the cellular context.

## Introduction

### The Nucleosome and the Histone H3 Family

Cell fate decisions are central to development, normal homeostasis, and responding to infections, injury, and aging. During these processes, stem cells sustain the ability to self-renew and differentiate. These stem cell properties are tightly controlled by signaling pathways that orchestrate complex transcriptional and posttranscriptional layers of gene regulation. The structural foundation of these cell type-specific transcriptional programs is determined by DNA-protein-RNA complexes within the nuclear space. In 1879, Walther Flemming first described this complex structure in mitotic salamander cells, terming it “chromatin” from the Greek word chroma, referring to the color affinity of the intensely stained nuclear content. Almost a century later, X-ray diffraction patterns of chromatin by Maurice Wilkins, Vittorio Luzzati, and Aaron Klug suggested a repeating building unit and that histones are involved in packaging DNA (Luzzati and Nicolaieff, 1959; Wilkins et al., 1959). Indeed, subsequent enzymatic digestion of chromatin isolated from rat liver cells using DNA nuclease revealed multiples of 200 base pair DNA fragments (Hewish and Burgoyne, 1973). Electron micrographs of chromatin fibers also revealed that these repeating units, known as nucleosomes, were composed of DNA wrapped around histone molecules (Kornberg, 1974; Olins and Olins, 1974; Oudet et al., 1975). Two decades later, Karoline Luger’s structural studies determined that the core of the nucleosome consists of 147 base pairs of DNA wrapped around an octamer of histones assembled from a tetramer of histone H3:H4 dimers that is flanked by two Histone H2A:H2B dimers (Luger et al., 1997). Since then, nucleosomes have become known as highly dynamic hubs of DNA-protein-RNA interactions, that not only allow for cell-type specific gene regulation, but for higher order chromatin organization important to many cellular processes.

The histones within the core nucleosome are interchangeable with different isoforms, identified as histone variants by Franklin and Zweidler (1977). While the repertoire of histones continues to expand, the Histone H3 family in particular has been in the spotlight of chromatin and cellular plasticity research. H3 carries the majority of well characterized heritable posttranslational modifications (PTMs) known to date, evolved a centromere specific histone variant, has a pronounced effect on nucleosome stability compared to other histones and can act as an oncogene due to mutations within critical residues subject to PTMs (Filipescu et al., 2014). Moreover, the current research on H3 shows how profound this integral nucleosome component is to the regulation of chromatin states and cell identity (Filipescu et al., 2014; Loppin and Berger, 2020; Martire and Banaszynski, 2020). The histone H3 family is composed of 8 members, H3.1, H3.2, H3.3, CENPA, H3.4, H3.5, H3.X and H3.Y. While the latter 4 members are poorly characterized, the replicative H3.1/H3.2 variants and the non-replicative H3.3 and CENPA variants have received much attention.

**FIGURE 1:**
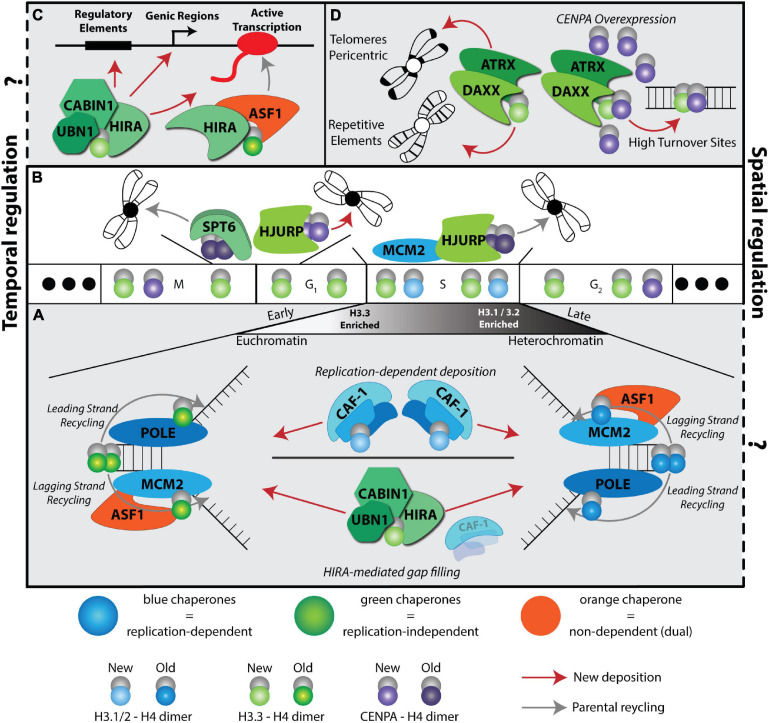
A complex histone chaperone network regulates spatiotemporal H3 deposition. The temporal H3 expression and deposition pathways are illustrated using the cell cycle phases (G1, S, G2, M) as a centerpiece. Three solid dots on the ends indicate continuous cycling. Histones H3.1/2 (blue) are expressed during S phase, H3.3 (green) is expressed throughout the cell cycle and CENPA is expressed during G2 and mitosis. Histone deposition molecules are color coded: RD (blue), RI (green), Dual (orange). Recycling of old histones (dark shaded H3 molecules) and deposition of new histones (light shaded H3 molecules) are indicated by gray and red arrows, respectively. The spatial deposition is illustrated either in the context of heterochromatin or euchromatin compartments, transcription sites or repetitive elements. **(A)** CAF-1 trimer deposits new H3.1/2 during S phase. ASF1 and MCM2 cooperate to promote histone recycling to the lagging strand, while subunits POLE3-POLE4 recycle histones to the leading strand. Early replicating regions occur in euchromatin and are enriched with H3.3 histones, while late replicating regions occur in heterochromatic regions and are enriched with replicative histone H3.1/2. In the absence of CAF-1, HIRA deposits H3.3 at replication sites in a gap-filling mechanism. **(B)** CENPA is deposited in a spatiotemporal manner. HJURP deposits new CENPA at centromeres during late M and early G1 phases. SUPT6 recycles parental CENPA during transcription in late M and early G1 to prevent CENPA eviction at centromeres. HJURP cooperates with MCM2 during S phase to recycle parental CENPA at the centromere. **(C)** HIRA-mediated H3.3 deposition at a transcriptional unit. Two different HIRA complexes deposit new and parental H3.3. **(D)** DAXX-ATRX deposits H3.3 at telomeres, pericentric regions, and repetitive elements. When CENPA is overexpressed, DAXX-ATRX deposits heterotypic tetramers containing both H3.3 and CENPA at sites of high histone turnover.

H3 histone forms differ markedly in their gene structure, expression profiles, deposition mode and post translational modifications (Mendiratta et al., 2018; Martire and Banaszynski, 2020). H3.1 and H3.2 are found in multiple copies in the genome. In dividing cells, they are defined as replicative histones due to their S-phase specific expression and replication-dependent deposition, which allows for chromatin assembly in the wake of DNA synthesis when parental histones are diluted (Figure 1A; Mendiratta et al., 2018; Grover et al., 2018). The H3.3 variant differs from the H3.1 and H3.2 by only 5 and 4 amino acids, respectively. On the other hand, there are two H3.3 genes in mammals, H3f3a and H3f3b, that encode identical amino acid sequence but are different in their primary DNA sequence and are tightly regulated transcriptionally and post-transcriptionally in different cell types (Muhire et al., 2019). H3.3 genes are expressed throughout the cell cycle in dividing cells (Figure 1) and are highly abundant, if not the predominant H3, in non-dividing cells. Finally, CENPA, the centromere specific H3 variant, shares less than 51% sequence identity with the replicative histones and forms a highly compacted nucleosome core that is wrapped by only 121 base pairs of DNA. It is encoded by one gene expressed during G2 and mitosis in preparation for new CENPA incorporation in centromeres (Figure 1B; Jansen et al., 2007; Martire and Banaszynski, 2020). For recent evolutionary analysis of H3 variants and their role in development and disease, readers are referred to (Buschbeck and Hake, 2017; Loppin and Berger, 2020).

Overall, the positioning, modifications, and histone composition of nucleosomes can have profound effects on chromatin accessibility to transcription factors at actively transcribed (euchromatic) and repressed (heterochromatic) compartments, whose activity determines cell identity. Indeed, recent integration of different epigenomic maps, including higher order chromatin structures, nucleosome positioning, histone distribution and modifications during early development, and different cell fate change paradigms, demonstrates the complexity of spatiotemporal chromatin rearrangements (Eckersley-Maslin et al., 2018; Pérez-Palacios and Bourc’his, 2018; Fang et al., 2018; Ishiuchi et al., 2021). However, how nucleosome components and assembly pathways contribute to this regulation is still being dissected.

### Histone Chaperone Roles in Nucleosome Dynamics and Beyond

Nucleosomes are diverse and dynamic. They can be shifted, assembled, or disassembled, and organized into different chromatin compartments through cooperation of histone chaperones, chromatin remodelers, and chromatin modifying factors (Dixon et al., 2012; Struhl and Segal, 2013). In particular, histone chaperones are the life partners of the nucleosome’s core histones. They are involved in escorting histones from their synthesis, storage, and transport, to histone modification, deposition, eviction, and recycling in the nucleosome (Grover et al., 2018).

Originally, the term “molecular chaperone” was used by Ron Laskey who isolated and characterized the function of nucleoplasmin as the first histone chaperone using Xenopus egg extracts (Laskey et al., 1977, 1978). This was the proof of principle that histone chaperones are involved in nucleosome assembly by binding directly to histones, neutralizing their positive charges and preventing non-specific interactions and aggregates formed with DNA *in vitro* under physiological salt concentrations (Dilworth et al., 1987). This seminal discovery laid the groundwork for our knowledge today that nucleosome assembly involves a step wise transfer of H3:H4 and H2A:H2B dimers and a complex network of histone chaperone partners (Hammond et al., 2017). As a whole, histone chaperones have no consensus sequences or structural motifs, making the discovery of novel chaperones more challenging. They exhibit considerable differences perhaps due to specialized functions in (1) recognition of distinct histone variants, (2) dedicated activities in different DNA transactions: replication, transcription, repair, and recombination, (3) diverse complex formation in histone dependent or independent manners and (4) spatiotemporal requirements in different cell-types.

**FIGURE 2:**
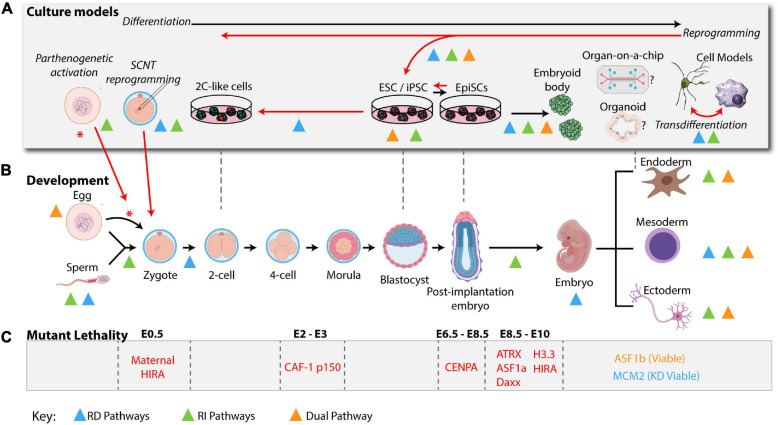
H3 deposition pathways in a physiological setting: development and culture models. H3 Deposition pathways characterized in cell fate transitions during development or in cellular systems are indicated by colored triangles using the same color scheme used in Figure 1 (RD: blue, RI: green, and dual: orange). **(A)** *Ex vivo* models are represented above their most relevant developmental stages as indicated in the developmental timeline (B) Black arrows indicate *differentiation* of the zygote from a higher potency to a lower potency. Red arrows indicate key cell plasticity pathways, including, zygote reprogramming following oocyte activation via *parthenogenesis* or *SCNT*, *reprogramming* of somatic cells to pluripotency and *transdifferentiation* of cells directly from one lineage to another. **(B)** A mouse developmental timeline, depicting the sperm and oocyte generating the zygote, early cleavage embryos, blastocyst, and early post-implantation embryo followed by specialized lineages discussed in the text (mouse development icons were created using BioRender software). **(C)** Summary of histone variants or histone chaperone mutant lethality in early embryo development. See Table 1 for a summary of phenotypes and corresponding references.

**FIGURE 3:**
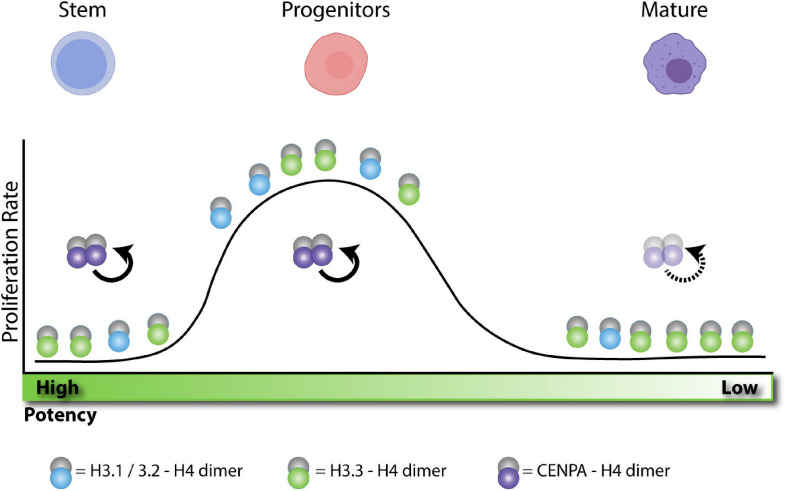
H3 enrichment depends on the proliferative state and cellular potency. Adult stem cells remain in a quiescent state. Without replication these cells become enriched in H3.3. After activation, stem cells differentiate into progenitors and rapidly expand, becoming more enriched in H3.1/2 histone. After expansion, progenitors terminally differentiate into post-mitotic cells. Without replication, these cells also become enriched in H3.3. In quiescent cells, CENPA is actively maintained at the centromere during transcription to preserve proliferative potential. During expansion, centromeres are maintained by HJURP and SUPT6 as indicated in Figure 1. After terminal differentiation, post-mitotic cell centromeres can lose stability over time.

Histone H3 and its variants especially exhibit a highly complex and specialized histone chaperone network in addition to more general chaperone interactions as seen with other histones (Figure 1). However, studying the interplay between the different histone chaperone pathways has been challenging to disentangle in the context of cell fate transitions. Recent technological advances combining histone labeling, genetic engineering, epigenomics, high resolution microscopy, and structural and biochemical approaches in different contexts have started to shed light on understanding the role of nucleosome dynamics in cell fate decisions. In this review, we focus on H3 deposition pathways in the context of the cell cycle and how they relate to cell fate transitions during early development and several culture systems (Figures 2, 3 and Table 1). The roles of other histone variants, accompanying chaperones, chromatin remodelers and modifiers in cell fate transitions are reviewed in recent publications, including this special issue.

**TABLE 1:** Histone and histone chaperone roles in cell fate decisions.

**Pathway**	**Protein**	**Function/ Phenotype**	**Mechanism**	**System**	**References**
RD	H3.1/2	Male fertility	–	Human/mouse sperm	Hammoud et al., 2009; Bush et al., 2013; Erkek et al., 2013; Tang et al., 2013, 2015; Jang et al., 2015; Das et al., 2017
		Zygote/embryo development	Replication-associated deposition (?)	Mouse embryo	Ishiuchi et al., 2021
RD	CAF-1	Hematopoiesis	–	Mouse bone marrow	Volk et al., 2018
		CD8 + T-cell identity	HDAC and LSD1 CD4 silencing in conjunction with DNMT3a	Mouse T-cell	Ng et al., 2019
		Restricts plasticity	Chromatin accessibility and heterochromatin maintenance	Mouse MEF/HSPC reprogramming, ESCs, B-cell to Mac transition, MEF to neuron	Cheloufi et al., 2015; rev. in Cheloufi and Hochedlinger, 2017
		Differentiation	H3K27me3 mediated silencing	mESCs	Cheng et al., 2019
		Heterochromatin organization	LSD1?	mESCs/embryo	Houlard et al., 2006
		Zygote/embryo development	Replication-associated deposition	Mouse embryo	Ishiuchi et al., 2015, 2021
RD	MCM2	Decidualization	Cell cycle arrest	Mouse endometrial stromal cells	Kong et al., 2016
		Symmetric cell division	Symmetric histone recycling	mESC/HeLa	Petryk et al., 2018; Ma et al., 2020
		Adult SC deficiency	DNA damage/replication deficiency (?)	Mouse *in vivo*	Pruitt et al., 2007
		CD8 + T-cell identity	–	T-Cells	Ng et al., 2019
RI	H3.3	Male fertility	–	Human/mouse sperm	Hammoud et al., 2009; Erkek et al., 2013; Das et al., 2017
		Fertilization	Sperm decondensation		
		Muscle Differentiation	MyoD/MEF2 expression through H3.3 deposition	C2C12 to myotube	Yang et al., 2011, 2016
		Osteoblast conversion	H3.3 deposition	C2C12 to osteoblast	Song et al., 2012
		Pluripotency	PRC2 Recruitment	mESCs	Banaszynski et al., 2013; Schlesinger et al., 2017
		MEF reprogramming	HIRA-mediated H3.3 deposition at promoters	MEFs/iPSC	Fang et al., 2018
		Transdifferentiation	–	MEFs/iHPs	Fang et al., 2018
		Differentiation	–	mESC to neuron	Fang et al., 2018
		Macrophage activation	H3.3S31ph SETD2 recruitment	mouse macrophage	Armache et al., 2020
		Differentiation	H3.3S31ph-mediated p300 activity and enhancer acetylation	mESC	Martire et al., 2019
RI	DAXX	Restricts plasticity	ERV Accessibility	Mouse pancreas	Wasylishen et al., 2020; Wu et al., 2020
		Neuron activation	Daxx-phosphorylation H3.3 deposition.	Mouse CNS	Michod et al., 2012
RI	HIRA	Fertility	rRNA Transcription; H3.3-deposition	Mouse zygote	Lin et al., 2014
		Muscle Differentiation	MyoD/MEF2 expression through H3.3 deposition	C2C12 to myotube	Yang et al., 2011, 2016
		Osteoblast conversion	H3.3 deposition	C2C12 to osteoblast	Song et al., 2012
		Adult hematopoiesis	Chromatin accessibility	Mouse bone marrow	Chen et al., 2020
		Cardiac differentiation	H3.3 deposition	mESC differentiation to cardiac	Dilg et al., 2016; Saleh et al., 2018
		Myofiber maintenance	–	Muscle mouse myocytes *in vivo*	Valenzuela et al., 2017
		Pluripotency/self-renewal	PHB/H3.3 deposition chromatin promoters	hESC	Zhu et al., 2017
		Mesoderm development	–	Mouse embryo	Roberts et al., 2002
		Neurogenesis	SETD1A-mediated beta-catenin regulation	Mouse CNS	Li and Jiao, 2017; Jeanne et al., 2021
RI	CENPA	Gametogenesis	CENPA retention	Sperm and oocyte	Rev in. Das et al., 2017
RI	HJURP	Cellular senescence	P53-dependent (?)	HDFs and HUVEC	Heo et al., 2013
		Cellular quiescence	centromere identity	human RPE1 and starfish oocyte	Swartz et al., 2019
Dual	ASF1A	Lineage differentiation	Histone eviction at promoters	Mouse EBs, neural differentiation	Gao et al., 2018
	ASF1A	Pluripotency/reprogramming	H3K56 acetylation	H9 ESCs, hADFs	Gonzalez-Muñoz et al., 2014
	ASF1A	Muscle differentiation	MyoD/MEF2 expression through H3.3 deposition	C2C12 to myotube	Yang et al., 2011
	ASF1A	Osteoblast conversion	H3.3 deposition	C2C12 to osteoblast	Song et al., 2012
	ASF1B	Cell proliferation	H3.3 recruitment	Human beta-cells	Paul et al., 2016
	ASF1B	Female fertility	–	Mouse oocyte	Messiaen et al., 2016

*Summary of histone or histone chaperone roles in cell fate decisions in context of the systems studied and illuminated mechanisms.*

## Histone H3 Deposition Pathways

### Spatiotemporal Regulation of CAF-1, HIRA and DAXX Pathways

Early work on nucleosome assembly pathways demonstrated a specificity of histone H3 chaperones to assemble nucleosomes in a DNA replication dependent (RD) or independent (RI) manner (Almouzni and Méchali, 1988; Smith and Stillman, 1989; Ray-Gallet et al., 2002). This cell cycle determinant of histone chaperone pathways is further complicated by the specific partnerships of histone chaperones with different variants, parental “old” histones versus newly synthesized histones and the deposition coordinates in the genome (Figure 1). Assembly of newly synthesized histones has been extensively studied and recent work is beginning to uncover the recycling mechanisms of old histones (Venkatesh and Workman, 2015;

Serra-Cardona and Zhang, 2018). Moreover, some plasticity and redundancy across these pathways has been observed, especially in terms of handing over new histones, recycling old histones, or when some pathways are absent or compromised (placeholding/gap filling) (Figures 1A,B) (Dunleavy et al., 2011; Ray-Gallet et al., 2011; Schneiderman et al., 2012).

The first discovery of a RD histone chaperone was reported more than three decades ago from human cell extracts by the purification and characterization of the Chromatin Assembly Factor complex CAF-1, a trimeric subunit composed of p150, p60 and Rbbp4 (Smith and Stillman, 1989). To date, CAF-1 is the only known histone chaperone that loads newly synthesized H3.1/2:H4 dimers onto DNA in a RD manner (Figure 1A).

It took another decade to discover the Histone H3.3-specific chaperone HIRA (histone cell cycle regulator). Subsequently, HIRA was found to also function as a trimeric complex with UBN1 and CABIN subunits (Lorain et al., 1998; Magnaghi et al., 1998). The HIRA complex incorporates newly synthesized H3.3 in a RI manner and was initially associated with histone deposition at active sites of transcription (Ahmad and Henikoff, 2002; Ray-Gallet et al., 2002; Figure 1C).

Genome wide distribution of H3.3 deposition in mouse embryonic stem cells (mESCs) led to the discovery of an additional H3.3-specific histone chaperone death domain associated protein (DAXX) (Tang et al., 2004; Goldberg et al., 2010; Lewis et al., 2010). DAXX in complex with the chromatin remodeler ATRX specializes in the deposition of H3.3 at repetitive elements, including telomeres, pericentromeric DNA, and a subset of endogenous retroviral elements (Goldberg et al., 2010; Lewis et al., 2010; Elsässer et al., 2015; Hoelper et al., 2017; Figure 1D). Although, the cell cycle timing of DAXX mediated H3.3 deposition is unclear, considering that the deposition of newly synthesized CENPA on centromeres via the Holliday junction recognition protein (HJURP) histone chaperone occurs in late mitosis/early G1 phase (Dunleavy et al., 2009; Figure 1B), it is tempting to speculate that the H3.3 deposition at repetitive elements coincides with this timing.

During S phase, HJURP mediates parental CENPA recycling with MCM2, a subunit of the helicase complex, and H3 histones act as place holders until new CENPA is deposited (Dunleavy et al., 2011; Zasadziñska et al., 2018; Figure 1B). Notably, CENPA overexpression leads to heterotypic deposition with H3.3 by the histone chaperone DAXX, reinforcing the plasticity of the histone chaperone network (Figure 1D; Arimura et al., 2014; Lacoste et al., 2014).

### The ASF1 Hub

The ASF1 histone chaperone, originally discovered in yeast (Tyler et al., 1999), serves dual RD and RI nucleosome assembly pathways as it functions in handing over newly synthesized H3.1:H4 and H3.3:H4 dimers to CAF-1 and HIRA complexes, respectively (Grover et al., 2018). In mammals, the ASF1 network is diversified by the emergence of two paralogs, ASF1A and ASF1B, with preferences for HIRA and CAF-1 p60, respectively (Tang et al., 2006; Abascal et al., 2013). Interestingly, recent work by Almouzni’s group demonstrated that ASF1 coordinates with HIRA to distinguish between old and new histone incorporation during transcription. In this model, ASF1 participates in the HIRA complex to recycle old H3.3, while new H3.3 is deposited via a UBN1-containing complex (Figure 1C; Torné et al., 2020).

Depletion of both ASF1 paralogs showed ASF1 is important for histone recycling during replication. This recycling is accomplished in partnership with MCM2, a subunit of the helicase complex. Together, they promote the recycling of old H3:H4 dimers in a RD manner (Groth et al., 2007; Huang et al., 2015; Figure 1A). Furthermore, recent evidence indicates MCM2 promotes symmetric loading of parental H3:H4 during DNA replication by preventing biased parental histone loading on the leading strand (Petryk et al., 2018). Conversely, newly identified histone chaperones POLE3-POLE4, subunits of the leading strand polymerase POLE, are proposed to load parental H3:H4 on the leading strand (Bellelli et al., 2018; Figure 1A). Considering these intriguing findings, it will be interesting to probe the interplay between POLE subunits, MCM2, and ASF1 and how leading and lagging strand deposition is balanced during self-renewal or differentiation.

Genome wide distribution and high-resolution microscopy mapping of parental histones in human cells demonstrate that H3.1 and H3.3 associate with late and early replicating regions, respectively (Clément et al., 2018; Mendiratta et al., 2018; Figure 1A). Therefore, it is tempting to hypothesize that due to its preference for HIRA interaction, ASF1A could act by recycling H3.3 while ASF1B could participate in recycling H3.1/2:H4 at the replication fork. It would therefore be interesting to investigate how ASF1 paralogs could participate in loading parental H3.1/2:H4 and H3.3:H4 dimers depending on their associated partners, replication sites/timing and leading versus lagging strand preference.

Another histone chaperone discovered in yeast, Suppressor of Ty 6 (SPT6) (Kaplan et al., 2003) plays a role in recycling parental H3:H4 during transcription and ASF1 can fill in the nucleosome gaps in spt6 yeast mutants (Jeronimo et al., 2019). Interestingly, yeast has only one form of H3 that is closely related to the mammalian H3.3 variant (Talbert and Henikoff, 2010). Given that SPT6 has an important role in transcription elongation (Vos et al., 2018, 2020), the interplay of ASF1 and SPT6 in histone deposition and recycling during transcription may yield further insights into nucleosome dynamics during this process. While commonly cited as a H3 histone chaperone, as shown in yeast, it remains to be concretely determined if SPT6 functions in H3:H4 deposition in mammalian cells. However, in support of this hypothesis, a recent study proposes a role for the histone chaperone SPT6 as a recycling factor for CENPA, with evidence in Drosophila and HeLa cells (Bobkov et al., 2020; Figure 1B).

Altogether the recent advances in labeling and mapping spatiotemporal distribution of old and new histones, structural mechanisms of histone-chaperone recognition, how they interact directly with DNA templates, and mapping nucleosome positions during different DNA processes have deepened our understanding on how the cell uses nucleosome assembly to maintain or reprogram chromatin organization. While mechanistic studies are largely performed *in vitro* or in immortal human or mouse cell lines, this knowledge could provide the fundamental mechanisms at play for stem cell maintenance and lineage commitment during development and tissue homeostasis (see sections below).

## H3 Deposition in Different Physiological Settings

How the nucleosome assembly pathways discussed above (Figure 1) modulate cell fate decisions and cell identity maintenance puzzled scientists for decades. The recent breakthroughs in the field coincide with a burst of technological advances and their relevant applications in studying culture models and developmental processes (Figure 2). A wide spectrum of phenotypes has been observed upon manipulation of RD and RI nucleosome assembly pathways that depend on spatiotemporal histone requirements, with RD pathways arguably more challenging to study due to their requirement in cellular proliferation and subsequent lethality. Here, we will describe some examples and discuss possible mechanisms along with future implications.

## Culture Models

Culture models to study cellular differentiation and reprogramming are powerful platforms to explore the molecular mechanisms orchestrated by the histone variants-histone chaperone network because they provide an opportunity to study cell autonomous effects within specific lineages, with some systems being more homogenous compared to others and are compatible with biochemical approaches (Figure 2A). Here we describe stem cell-based systems that mimic normal development and that have proved useful in understanding H3 deposition pathways.

### Embryonic Stem Cells

ESCs are the earliest embryonic cells that can be captured *in vitro* from the blastocyst and propagated without compromising their pluripotent potential (Figure 2A; Evans and Kaufman, 1981; Martin, 1981). A change in culture conditions and/or intrinsic factors can coax ESCs to interchange their potency levels and/or commit to different lineages (Figure 2A). For example, ESCs can be maintained in culture in various states of pluripotency reflecting naïve (ground) or primed developmental states. Human ESCs (hESCs) derived from blastocysts reflect an even later pluripotency state during mouse development akin to the epiblast stem cells (epiSCs) that can be derived from the post implantation mouse embryo (Figure 2A).

In contrast to the developmental arrests that have been observed in CAF-1 depleted embryos (Houlard et al., 2006), it has been more amenable to probe the function of H3 deposition pathways particularly in mESCs without compromising cellular viability. The loss of the Chaf1a and Chaf1b subunits of the CAF-1 complex in ESCs results in their reprogramming to an earlier embryonic cell state mimicking the two-cell stage of embryonic development (2C-like cells) (Ishiuchi et al., 2015, 2021). The conversion of ESCs to 2C-like cells is dependent on cell progression through S-phase and on the chromatin assembly activity of CAF-1, displaying similar molecular features to spontaneously derived 2C-like cells and 2-cell stage embryos. Although, the recent finding that H3.3 deposition resumes non-canonical distribution upon CAF-1 ablation in ESCs suggests that this 2C-like cell fate induction is in fact reflecting a transient or even earlier embryonic cell state (Ishiuchi et al., 2021; Table 1). It will be interesting to compare additional epigenetic features and transcriptomes during this developmental window in the CAF-1 mutants.

This induction of a permissive chromatin state upon CAF-1 loss in ESCs is consistent with the initial observations where CAF-1 loss in ESCs affects heterochromatin features specific to stem cells (Houlard et al., 2006). Indeed, recent work demonstrated that CAF-1 loss impairs ESC differentiation in an embryoid body assay (Cheng et al., 2019; Figure 2A). Interestingly, this defect was linked to failure of establishing H3K27me3 marks at pluripotency promoters upon differentiation through CAF-1-PCNA and CAF-1-Polycomb (PRC2) recruitment to the replication fork. In this context, Cheng et al. (2019) detected a reduced association of H3.1 and H3K27me3 with replicating chromatin. It would be interesting to test how H3.1/H3.3 ratio affects H3K27me3 establishment and how parental histone inheritance is influenced during this process, potentially conferring a resistance of CAF-1 ESCs to differentiation.

Parental and new histone distribution was examined at a single cell level using a Wnt3a-induced asymmetric ESC division model, demonstrating there is differential distribution of old and new canonical histones in the daughter cells (Ma et al., 2020). This suggests a specialized action of histone chaperones during asymmetric division. Additionally, in light of the new implication that MCM2 promotes symmetric cell division through RD histone recycling to the lagging strand (Bellelli et al., 2018; Petryk et al., 2018), possibly with ASF1 as seen in HeLa cells (Bellelli et al., 2018; Petryk et al., 2018; Figure 1A), it will be interesting to examine how perturbation of different histone chaperones in ESC self-renewal and differentiation affects histone distribution during replication. Of note, the involvement of CAF-1 and histone mutations has been previously proposed to play a role in asymmetric histone deposition during *C. elegans* development (Nakano et al., 2011). Whether similar mechanisms are conserved in mammals remains to be tested.

Contrary to the loss of CAF-1 in ESCs, perturbation of RI nucleosome assembly in ESCs does not alter gene expression profiles nor compromise ESC identity under self-renewal conditions. However, H3.3 together with its partners DAXX and ATRX are involved in silencing repetitive elements in ESCs, including a subset of retroelements and telomeres (Goldberg et al., 2010; Lewis et al., 2010; Elsässer et al., 2015; Hoelper et al., 2017; Figure 1D). Remarkably, this effect is more pronounced in more naïve or hypomethylated ESC cultures reflecting an important role in early preimplantation development (He et al., 2015). Whether this effect is purely a function in safeguarding genome stability or fine tuning transcriptional programs co-opted by repetitive elements remains to be explored (Macfarlan et al., 2012). The physiological effect of RI nucleosome assembly pathway depletion is exacerbated upon differentiation of ESCs where lineage specific gene expression programs are perturbed.

Interestingly the loss of ASF1A, HIRA and H3.3 affect Histone H3 K27 methylation (H3K27me3) specifically at developmentally regulated genes (Banaszynski et al., 2013; Gehre et al., 2020; Gao et al., 2018; Figure 2A and Table 1). HIRA dependent deposition of H3.3 is proposed to establish bivalent marks in ESCs at developmentally regulated genes while ASF1A dependent disassembly of nucleosomes facilitates resolution of bivalent domains upon ESC differentiation. H3.3 loss in ESCs also reduces enhancer H3 acetylation marks including H3K27ac, H3K18ac, H3K64ac, and H3K122ac (Martire et al., 2019). H3K27ac, in particular, a mark known to coincide with active enhancers is stimulated by the phosphorylation of the serine 31 residue on the H3.3 tail in mESCs. H3.3 serine 31 (H3.3S31) is one of the amino acids unique to H3.3. Supplementing H3.3 KO mESCs with replicative histone H3.2, bearing an alanine 31 residue, cannot rescue the enhancer acetylation defect despite being deposited at these sites. Moreover, the loss of H3.3 in ESCs does not affect chromatin accessibility or the recruitment of p300 histone acetyltransferase at enhancer elements suggesting that the H3.3S31 residue is uniquely required downstream of HIRA mediated deposition for subsequent chromatin signaling pathways. Consistent with the loss of H3K27me3 or DNA methylation in ESCs, the reduced acetylation of the H3.3 KO is tolerated by ESCs under self-renewing conditions with no dramatic effect on gene expression (Martire et al., 2019). However, their differentiation triggers defects in chromatin accessibility and establishing active enhancer elements and subsequent activation of differentiation genes.

A recent systematic characterization of all four H3.3 specific residues in a Xenopus gastrulation model reinforces the essential role of H3.3S31 specific phosphorylation during this developmental process (Sitbon et al., 2020). Strikingly, the replacement of all three H3.3 residues that are required for specific RI chaperone interactions with their H3.2 replicative counterparts was compatible with normal gastrulation. It will be interesting to perform similar genetic analyses in the context of ESC differentiation.

Recent work interrogated the function of H3.3 lysine residues (K4 and K36) in ESCs (Gehre et al., 2020). Alanine substitutions of H3.3K4 and H3.3K36 did not compromise ESC self-renewal but perturbed lineage specific transcriptional programs and differentiation, albeit with varying degrees. H3.3K4, but not H3.3K36, mutant ESCs exhibited severe defects and resulted in reduced H3.3 deposition at regulatory elements, especially promoters, independently of the lysine charge. While wild type replicative histones share these same residues with H3.3 and are able to compensate and maintain normal nucleosome density around transcription start sites (TSS), this is not sufficient to maintain the correct chromatin state. This observation reinforces the importance of H3.3 specific residues. Interestingly, H3.3K4 mutation did not perturb H3.3 histone chaperone expression or binding but diminished the interactions with chromatin remodelers and increased RNA polymerase activity. The authors thus propose a role for K4 in maintaining H3.3 at regulatory elements through proper recruitment of remodelers and accurate transcriptional activity. This study highlights how histone chaperones act in concert with remodelers and accompanying PTM signals to regulate nucleosome dynamics.

Taken together, these H3.3 studies in ESCs and model organisms justify some of the needs to incorporate H3.3 at regulatory elements and highlight the relevance of unique and common H3 residues in regulating nucleosome dynamics and setting specialized chromatin environments post nucleosome assembly (Figure 2 and Table 1).

Considering these findings, it is tempting to speculate that during mESC differentiation, RD assembly pathways play a passive role in diluting ESC identity and RI pathways play an active role in establishing new identity. However, discrepancies in the effect of manipulating these pathways between hESCs compared to mESCs still need to be resolved. For example, the loss of both HIRA and ASF1 compromise hESCs self-renewal (Gonzalez-Muñoz et al., 2014; Zhu et al., 2017). HIRA loss in hESCs results in downregulation of pluripotency factors, activation of various lineage markers and differentiation. Moreover, in hESCs, the HIRA complex is proposed to associate with a stem cell specific subunit PROHIBITIN that stabilizes distinct complexes and cooperates with HIRA to regulate the metabolic circuitry in hESCs through H3.3 deposition. Considering that hESCs resemble mouse epiSCs (Figure 2A), which reflect a more primed pluripotent cell state, it is possible that phenotypes similar to mESCs could arise when examined in more naïve hESCs (Brumbaugh et al., 2019). It would be exciting to probe histone exchange dynamics and histone chaperone networks during interconversion of these pluripotency states to build on the current study documenting the changes in histone modifications to shape chromatin environments (De Clerck et al., 2019).

### Reprogramming and Transdifferentiation

Reprogramming and transdifferentiation platforms have proved valuable in revealing unprecedented physiological roles of nucleosome assembly pathways in somatic cells (Figure 2A). For example, probing the function of CAF-1 in the context of transcription factor mediated reprogramming of mouse embryonic fibroblasts to induced pluripotent stem cells (iPSCs) implicated its role in maintaining somatic cell identity. In this system, CAF-1 is proposed to act in part through its nucleosome assembly function by restricting access to pluripotency transcription factors (Cheloufi et al., 2015; Cheloufi and Hochedlinger, 2017). Supporting the role of CAF-1 in reprogramming, CAF-1 depletion in mESCs facilitates the generation of cloned blastocysts using somatic cell nuclear transfer technology and transdifferentiation between different lineages (Cheloufi et al., 2015; Ishiuchi et al., 2015). Contrary to CAF-1, ASF1A loss inhibits reprogramming of human somatic cells to iPSCs (Gonzalez-Muñoz et al., 2014). In this system, ASF1A co-expression with pluripotent transcription factor OCT4 is sufficient to reprogram human adult dermal fibroblasts when exposed to the oocyte-specific paracrine growth factor GDF9. In this context, ASF1A is proposed to work by promoting acetylation of histone H3K56 and cooperating with OCT4 to activate the pluripotency transcriptional network. ASF1A acts upstream of CAF-1 as a donor of newly synthesized histones but its functions also overlap with other nucleosome assembly pathways (Figures 1A,C). Thus, this discrepancy in reprogramming phenotypes between CAF-1 and ASF1A can be purely dependent on the spatiotemporal requirement of histone deposition and/or histone chaperone-independent functions. The implication of ASF1A in cellular reprogramming stemmed from it being a maternally deposited factor in the oocyte cytoplasm. Similarly, the H3.3 histone variant proved to be an essential maternal factor for reprogramming and the development of fertilized, parthenogenetically derived and SCNT embryos (Wen D. et al., 2014) (Figure 2A). In this context, H3.3 plays an important role in nucleosome remodeling in either the parental pronuclei or the donor nucleus (Figure 2A). Consistent with a spatiotemporal requirement of histone deposition pathways in shaping cellular identity, a recent study demonstrates a dual role of HIRA mediated H3.3 deposition in maintaining somatic cell identity and establishing pluripotency during reprogramming (Fang et al., 2018). Thus, this global rearrangement of H3.3 deposition akin to the one observed during oogenesis and the early cleavage embryo represents an important mechanism in preparation for cell fate conversions (see preimplantation development & Ishiuchi et al., 2021). However, the interplay with other histone chaperone pathways remains to be determined especially in a setting where the cell cycle is required for cell fate switches.

In light of these observations, we propose that nucleosome pathways at different potency states during development can dictate cell identity maintenance versus cell fate commitment or reprogramming toward different lineages. This could be purely dependent on specific remodeling of histone variants distribution and cell cycle properties (Figure 3).

## From Gametogenesis to Early Embryonic Development

### Gametogenesis

The sperm and oocyte are highly specialized cell types that transmit both genetic and epigenetic information through generations (Figure 2B). During spermatogenesis, the genome undergoes a stepwise replacement of histones with transition proteins and ultimately protamines to form the highly condensed nucleus of the sperm (Raja and Renkawitz-Pohl, 2005; Torres-Flores and Hernández-Hernández, 2020). This process is thought to prevent DNA damage, confer better sperm quality, and reprogram the paternal nucleosomes in preparation for fertilization as protamine knockouts result in defective sperm and developmental arrest (Cho et al., 2001, 2003). The nuclear condensation within the sperm head is accompanied with complex PTMs of the disassembled histones and the newly deposited protamines which could potentially involve the action of different histone chaperones whose identity remains to be determined. However, despite the removal of nearly 90% of all histones in the sperm, CENPA is retained. Also, select nucleosomes at regulatory DNA elements retain H3.1/2 and H3.3 (Hammoud et al., 2009; Erkek et al., 2013; Das et al., 2017). The retention of nucleosomes containing specific histone variants and corresponding PTMs on the paternal genome is thought to be a mechanism for transmitting epigenetic information to the embryo (Champroux et al., 2018). Of note, profiling the accurate histone distribution in the sperm nucleus has proved to be technically challenging depending on the method used to purify mature sperm that have undergone proper histone replacement and chromatin digestion for histone pull downs (Yoshida et al., 2018).

Consistent with the histone retention in the mature sperm, genetic studies support these observations. To date, several mouse knockout and conditional alleles of the two H3.3 genes have been generated albeit with variable phenotypic consequences on the germline and embryonic development (see post-implantation development) possibly due to the different targeting strategies, genetic heterogeneity of the mouse strains as well as possible redundancy with testis specific H3 variants. For example, in a mixed C57BL/6 and 129 mouse background, H3f3a+/−; H3f3b−/− compound mutant with one remaining copy of the H3f3a gene are male sterile (Jang et al., 2015) while other studies reported that the surviving single H3f3a and H3f3b knockouts have variable levels of sterility (Couldrey et al., 1999; Bush et al., 2013; Tang et al., 2013, 2015; Yuen et al., 2014). Regardless of these differences, accumulating evidence supports a unique role of H3.3 in chromatin remodeling in the male germline.

The effect of H3.3 loss in the female germline is more debatable. In contrast to previous studies reporting female sterility of single H3.3 knockouts, H3f3a+/−; H3f3b−/− compound mutant females are viable and fertile (Bush et al., 2013; Jang et al., 2015; Tang et al., 2013, 2015; Figure 2B). This is surprising given that mature oocytes are devoid of replicative histones in their genome and that there is H3.3 redistribution during oogenesis in preparation for embryogenesis (Ishiuchi et al., 2021) (see preimplantation development). Furthermore, the requirement of H3.3 histone partners during gametogenesis warrant further investigations. Interestingly, Asf1b knockout mice are viable but have reduced reproductive capacity showing a more severe defect in females versus males (Messiaen et al., 2016). This study showed that ASF1B is specifically expressed in the female gonads during development and propose its role in regulating meiotic entry. In light of these findings and the proposed molecular function of ASF1 (see The ASF1 Hub), it is tempting to speculate that the Asf1b paralog plays a specialized role in retaining H3.3 containing nucleosomes in a RD manner in the oocytes. On the other hand, given that it does not have an effect on the male germline, it might function independent of its histone chaperone role.

Notably, due to the early lethality of most histone chaperone mutants (see Preimplantation Development), the use of conditional knockouts and the development of *ex vivo* gametogenesis culture systems (Hamazaki et al., 2021) will be instrumental in resolving these limitations. Furthermore, this will shed light on the mechanisms and differences in spatiotemporal H3 re-distribution during spermatogenesis and oogenesis and how germline reprogramming may impact epigenetic inheritance.

### Preimplantation Development

The fertilization between the sperm and oocyte gives rise to the zygote. In the zygote, both the paternal and maternal genomes undergo dramatic reprogramming events to give rise to the most plastic embryonic cell state known as “totipotency” (Figures 2A,B). If successful, the zygote will ultimately give rise to all cell types necessary for the development of an organism including the extraembryonic tissues. During this process, both the paternal and maternal pronuclei undergo major chromatin remodeling using maternally deposited factors in preparation for the first mitotic divisions and the transition to zygotic transcription (Figure 2B; Probst and Almouzni, 2011; Eckersley-Maslin et al., 2018). Accumulating evidence supports the idea that maternally deposited histones and histone chaperones are essential for reprogramming the zygote following fertilization. Indeed, the paternal genome is decondensed when incorporation of maternally deposited H3.3 replaces protamines, allowing for genome reprogramming (Loppin et al., 2005; Torres-Padilla et al., 2006). This is now known to be triggered by site specific phosphorylation of protamines by the RNA splicing factor SRPK1 which permits recruitment of nucleoplasmin (NPM2) and HIRA for protamine unloading and H3.3 deposition, respectively (Gou et al., 2020).

The manipulation of maternally deposited factors in oocytes followed by natural fertilization, parthenogenetic activation, or somatic cell nuclear transfer has been instrumental in understanding the mechanisms of RI incorporation of H3.3 onto parental chromatin (Figure 2B and Table 1; Lin et al., 2014; Wen D. et al., 2014). For example, deletion of HIRA in mouse oocytes results in inhibition of nucleosome assembly in the male genome and oocytes are unable to develop parthenogenetically. This study links HIRA-dependent H3.3 deposition to active transcription of ribosomal RNA in the zygote (Lin et al., 2014).

It will be interesting to probe the function of other H3.3 mediated site-specific histone chaperone pathways in the oocytes. Using ultra-low input native CHIP-seq a recent study generated a spatiotemporal map of H3.3 distribution during oogenesis, the zygote, and the early cleavage stage embryos (Ishiuchi et al., 2021). Interestingly, H3.3 undergoes a gradual global rearrangement during oogenesis forming a unique non-canonical pattern in the mature oocyte and zygote. At this developmental window, H3.3 is more broadly distributed across the genome and exhibits some enrichment at heterochromatic regions. Remarkably, the non-canonical H3.3 distribution is similar between the maternal and paternal pronuclei in the zygote but is different from other post-mitotic cells, such as neurons. Interestingly, this unique chromatin incorporation of H3.3 in the oocyte and zygote coincides with previously reported distinct epigenetic features, including chromatin accessibility, histone marks and DNA methylation during preimplantation development (Eckersley-Maslin et al., 2018; Burton et al., 2020). For example, temporal regulation of histone methyltransferases (SUV39H1&H2) involved in the deposition of the H3K9me3 repressive mark post fertilization results in establishing an accessible and non-repressive constitutive heterochromatin in the zygote that ultimately matures in later stages to a compacted and repressive state. While Ishiuchi et al., 2021 did not report a correlation between H3.3 deposition and H3K9me3 profiles in the zygote, it is tempting to speculate that this early marking of heterochromatin is established as a consequence of the slight preferential loading of H3.3 on heterochromatin compartments in the zygote. H3.3 deposition within these domains could create a chromatin environment to recruit SUV39H1 similar to the mechanism proposed for PRC2 recruitment in ESCs at developmentally regulated genes (Figure 2 and Table 1; Banaszynski et al., 2013). However, comparison of existing ChIP-seq data over constitutive heterochromatin domains may be challenging due to variable chromatin fragmentations, timing of the embryos, and considering multi-mapping reads at repetitive elements. Notably, as the zygote transitions to the 2-cell stage, the broad H3.3 distribution is reprogrammed to a more localized pattern reminiscent of the known canonical pattern initially described in ESCs. The reorganization and/or retention of H3.3 in the zygote occurs with the loading of replicative H3.1&H3.2 in a RD and transcription independent manner. Furthermore, it is regulated by CAF-1 as injecting a dominant negative form of the CAF-1 p150 subunit in the zygote reduces the canonical H3.3 rearrangement. Importantly this unique H3.3 rearrangement and deposition of replicative histones is essential for development as inhibition of maternally deposited CAF-1 results in developmental arrest at the 4-cell stage, consistent with previous reports ablating CAF-1 in the embryo (Figure 2C and Table 1; Houlard et al., 2006; Akiyama et al., 2011).

Considering the oocyte is devoid of replicative histones and that the expression of H3.1/2 peaks only after the embryo has undergone one cell division, it will be interesting to investigate how the non-canonical H3.3 distribution is propagated during the first round of replication, and how its recycling is regulated by histone chaperones in the context of replication timing. Therefore, it will be important to further characterize the histone chaperones required for H3.3 nucleosome exchange during these early cell divisions and how they are involved in preparing for zygotic genome activation and the establishment of heterochromatin and euchromatin domains. We think that the H3.3 broad distribution in the oocytes and zygote is possibly pre-programmed because of the need for fast RI eviction of protamines genome wide in the paternal pronucleus to ensure near-equal reprogramming of parental pronuclei in the zygote.

### Post-implantation Development

In contrast to the severity of maternal or zygotic CAF-1 loss, mice lacking ASF1A survive to mid-gestation (Figure 2C; Hartford et al., 2011). Given that Asf1b knockout mice are viable, it would be interesting to probe phenotypic consequences of Asf1a/b double knockouts. Similarly, in mice lacking either H3.3 gene and their histone chaperone partners, HIRA or DAAX, embryos also progress to mid-gestation (Figure 2C; Michaelson et al., 1999; Roberts et al., 2002; Jang et al., 2015). Interestingly, H3f3a/b double knockout mice progress through early patterning of the embryo but are lethal 2 days after implantation (Figure 2C). Single knockouts of H3f3a and H3f3b, however, are reported with a spectrum of phenotypes depending on the study resulting in compromised viability and sterility (see gametogenesis). Similarly, CENP-A knockout mice are also lethal shortly after implantation (Howman et al., 2000). Of note, chromatin defects in these different mutants suggest a convergent mechanism between RD and RI pathways where heterochromatin structures are primarily affected leading to mitotic defects and developmental arrest. The relatively late phenotypic manifestations of RI nucleosome assembly pathways could be due to maternal deposition of mRNA and proteins of histones and histone chaperones, or redundancy of histone chaperone pathways. It would therefore be interesting to test the effects of their maternal contribution and the consequences of individual histone chaperone perturbation on histone deposition and chromatin accessibility in the early embryo.

While examining histone exchange and chromatin dynamics in the early embryo remain challenging, current developments in CRISPR CAS9 gene editing (Adli, 2018; Anzalone et al., 2020), chromatin profiling technologies such as ATAC-seq, CUT&RUN and CUT&Tag that rely on a small number of cells (Buenrostro et al., 2013; Corces et al., 2017; Kaya-Okur et al., 2019; Meers et al., 2019), single cell multi-omics (Pérez-Palacios and Bourc’his, 2018), and culture systems are instrumental in understanding the mechanisms of nucleosome assembly during these most plastic cell states during development.

## Lineage Specific Differentiation, a Mix of in vivo and Culture Systems

Given the early lethal phenotypes of the H3 deposition pathways during development (Figures 2B,C), it has been challenging to probe their function in normal homeostasis. However, recent work using lineage specific differentiation systems as well as cancer and injury models (Evano et al., 2020) has not only shed light on how some mechanisms described above (Figure 1) are at play, but also associates histone chaperones with histone deposition independent functions. We postulate that these differences could be intimately linked to the spatiotemporal expression of the H3 deposition machinery and the specific cell cycle properties (e.g.: short versus long) within different lineages (Figure 3). Evidence so far suggests an important role of both RD and RI pathways in lineage restriction and maintenance. For example, CENPA in quiescent cells is specifically maintained to preserve proliferative potential (Figure 3; Swartz et al., 2019). Additionally, many cellular differentiation paradigms implicate CAF-1 and HIRA as a transcriptional repressor or activator, respectively (Figure 2B and Table 1).

Volk et al. demonstrated that while complete loss of CAF-1 in the mouse inhibits normal hematopoiesis, its reduced levels is tolerated (Volk et al., 2018). Low levels of CAF-1 protect the mice from cancer progression by triggering differentiation of MLL/AF9 leukemic cells into mature myeloid cells. In this setting, CAF-1 is proposed to maintain leukemic cell identity via its RD nucleosome assembly activity as well as its competitive binding to sites of myeloid specifying transcription factors.

In a screen for chromatin regulators, silencing the CD4 gene in CD8 + cytotoxic T cells, CAF-1 was also identified as a transcriptional repressor among other fork components, including MCM2 (Ng et al., 2019). In this setting, CAF-1, in addition to its nucleosome assembly function, is proposed to cooperate with DNA and histone modifying enzymes by binding directly to histone deacetylase and histone demethylases to ensure heritable silencing of the CD4 gene. More recently, single cell profiling demonstrates that CAF-1 loss in myeloid progenitor cells triggers their partial differentiation leading to a mixed cellular state (Preprint, Guo et al., 2020). Interestingly, in comparison to normal myeloid differentiation, CAF-1 loss triggers a unique chromatin accessibility environment and activation of multi-lineage specific transcription factors. How the transcriptionally repressive role of CAF-1 in these systems is linked to its H3 deposition, alternative deposition of histone variants and/or recruitment of chromatin regulators remain to be determined.

In contrast to CAF-1, HIRA is widely studied in various cellular models including several mesoderm-derived tissues as well as during neurogenesis (Figure 2B and Table 1). For example, during normal hematopoiesis, Chen et al. (2020) demonstrated that upon conditional deletion of HIRA in the mouse, long term hematopoietic stem cell (LT-HSC) function is impaired, leading to lethality. Interestingly, LT-HSC is thought to be in a more quiescent state. Accordingly, HIRA deletion had no effect in fetal hematopoiesis where hematopoietic stem cells are actively cycling. As seen with the mature oocyte and the early zygote, it is tempting to speculate that H3.3 deposition by HIRA could be involved in maintaining a unique chromatin environment in LT-HSCs that is subsequently remodeled during their self-renewal and differentiation (see model in Figure 3). HIRA is also required to maintain leukemic cells. Majumder et al. (2019) show that down-regulation of HIRA in chronic myeloid leukemia leads to a differentiation phenotype and implanting HIRA KO progenitors results in increased megakaryocyte differentiation. In this context, depletion of HIRA causes enrichment of H3.3 at promoters of key megakaryocyte differentiation factors GATA2 and MKL1, and a loss of H3.3 at erythroid differentiation promoters. It will be intriguing to dissect the mechanisms of differential H3.3 deposition upon HIRA loss. In addition to hematopoiesis, HIRA and ASF1 have been implicated in C2C12 myoblast cellular plasticity. Both HIRA and ASF1 are important for myoblast differentiation into myotubes and for their osteogenic conversion (Yang et al., 2011; Song et al., 2012). In myoblast differentiation HIRA and ASF1 drive MyoD expression and allow for H3.3 accumulation at critical enhancer regions. Additional studies implicate the role of HIRA in neurogenesis showing that it can interact with B-catenin to promote neurogenesis (Li and Jiao, 2017). In addition, *in vivo* conditional deletion of HIRA causes widespread defects in neurogenesis (Jeanne et al., 2021). Taken together in these systems, the HIRA mediated H3 deposition mechanisms are poorly characterized.

Interestingly, a recent study highlights HIRA’s gap filling mechanisms in the context of metastatic transformation of breast and colon cancer tissues (Gomes et al., 2019) (Figure 1A). In this setting, the activation of epithelial-to-mesenchymal transition genes to promote metastasis is dependent on downregulation of canonical H3 deposition of CAF-1 and HIRA-mediated H3.3 deposition at regulatory sites. It will be interesting to determine the mechanism underlying the specific deposition of H3.3 at these sites. Given HIRA’s selective deposition of H3.3 to regulatory elements and the body of active genes, HIRA’s function as a histone chaperone in addition to H3.3S31 specific phosphorylation could create a chromatin environment to facilitate the binding of transcription factors and chromatin regulators to maintain cell identity or instruct cell fate change depending on the cellular context and environment. Interestingly, H3.3 is required for neuronal stem cell proliferation and differentiation via promoting H3K16 acetylation. Whether H3.3S31 is required in this context remains to be explored (Xia and Jiao, 2017).

Another example of H3.3 guided recruitment of chromatin factors was recently highlighted while dissecting the transcriptional response to pathogens. In this context, selective phosphorylation of H3.3S31 at rapidly induced genes triggers a chromatin signaling cascade via recruiting a histone methyltransferase that promotes transcriptional elongation and repulsing a chromatin reader that inhibits transcription (Thorne et al., 2012; Guo et al., 2014; Wen H. et al., 2014; Armache et al., 2020).

Considering other H3.3 deposition pathways, conditional deletion of DAXX in pancreatic tissues supports its role in ERV silencing (Figure 2B and Table 1; Wasylishen et al., 2020). While no phenotypes were observed, the more permissible transcriptional state is proposed to increase responses to stressors and to impair recovery. Outside of DAXX function at repetitive elements, another study in neurons reported a non-canonical DAXX mediated H3.3 deposition at regulatory elements that is linked to neuronal activation (Michod et al., 2012). Furthermore, DAXX is responsible for the ectopic deposition of overexpressed CENPA which is a hallmark of many cancers (Figure 1D; Sharma et al., 2019). It will be interesting to probe how DAXX responds to the loss of HIRA in these systems and investigate the function of other histone chaperone mediated deposition of H3, including ASF1A/B, HJURP, SPT6 in these cellular settings.

## Discussion

Histone chaperones are in place to modulate the deposition of histones at the right place and right time and coordinate the action of accompanying chromatin factors, including lineage-specific transcription factors during quiescence, stem cell self-renewal, differentiation, or reprogramming. While the expression of histone variants during the cell cycle and development is well documented, the activity, complex diversity, and interplay of histone chaperones during these processes is poorly understood, especially in the context of cell fate transitions. This is clearly complicated by the multifunctional characteristics of histone chaperones as they play additional roles independent of nucleosome assembly that are in turn linked to chromatin regulation.

The lessons that we learned from studying H3 deposition pathways in the context of normal development, culture model and disease state suggest that the RD and RI H3 deposition pathways act in a balanced manner to maintain lineage identity and instruct cell fate change in response to signals. Aside from the traditional culture models, it will be interesting to exploit newly developed organoid culture models and gametogenesis platforms to characterize the mechanisms of histone exchange and apply the lessons we learned from model organisms (Figure 2A). These emerging culture models provide unique systems to perform biochemical studies and create high resolution spatiotemporal maps of histone deposition in the context of cell fate determination. Finally, future therapeutic avenues include (1) the identification of unique histone deposition machinery in disease states and investigating the epigenetic addictions as a consequence of histone mutations or compromised histone chaperone activity and (2) manipulate histone chaperone pathways to generate specific cell types for regenerative purposes.

## Author Contributions

SC proposed the topic of the review and outlined the structure. SC and JM mentored RF throughout the analysis and discussions. SC, JM, and RF reviewed the literature, wrote the review, designed the figures, and compiled the table. All authors contributed to the article and approved the submitted version.

## Conflict of Interest

The authors declare that the research was conducted in the absence of any commercial or financial relationships that could be construed as a potential conflict of interest.
